# Telephone hotlines for infectious disease outbreaks in Africa: A review and qualitative study

**DOI:** 10.4102/jphia.v15i1.608

**Published:** 2024-07-23

**Authors:** Noah T. Fongwen, Almighty Nchafack, Kyeng M. Tetuh, Jason J. Ong, Joseph D. Tucker, Gwenda Hughes, Rosanna Peeling

**Affiliations:** 1 Department of Diagnostics Access, Africa Centres for Disease Control and Prevention, Addis Ababa, Ethiopia; 2 Department of Clinical Research, Faculty of Infectious and Tropical Diseases, London School of Hygiene and Tropical Medicine, London, United Kingdom; 3 Department of Surveillance, Africa Centres for Disease Control and Prevention, Addis Ababa, Ethiopia; 4 Central Clinical School, Monash University, Melbourne, Australia; 5 Department of Global Health and Infectious Diseases, University of North Carolina, Chapel Hill, United States of America; 6 UK Rapid Support Team, London School of Hygiene and Tropical Medicine, London, United Kingdom

**Keywords:** mHealth, telephone hotlines, outbreaks, barriers, facilitators, sustainability

## Abstract

**Background:**

Mobile health interventions like telephone hotlines face challenges that may threaten their use, adoption and sustainability in Africa.

**Aim:**

We sought to understand the barriers and facilitators for sustainability of telephone hotlines used in infectious disease outbreaks in Africa using a scoping review and a qualitative study.

**Setting:**

Participants form 12 African countries and Database searches.

**Methods:**

Databases were searched for articles on the barriers and/or facilitators in operating telephone hotlines for outbreaks in Africa. One-on-one interviews and focus group discussions with 30 participants from 12 African countries were also conducted. Emerging themes from the review and interviews were identified and synthesised to focus on barriers and facilitators for the sustainability of the hotlines.

**Results:**

The search identified 1153 citations, and 25 studies were finally included. The articles were from 20 African countries. The government was the main source of funding in four countries. Barriers with calls and data management were the most frequent. Human resource barriers such as limited staff, high staff turnover, a lack of incentives and motivation were also significant. Financial barriers were the high cost of operation and huge dependence on external funders. Technological and infrastructural hurdles included limited Internet and phone coverage, malfunction and a lack of interoperability of software. Transitioning to either complete or shared government ownership with diversification and integration of the hotline into routine use was the main facilitator for sustainability.

**Conclusion:**

Strengthening technical capacity in telephone hotlines and ensuring financial sustainability are critical. Increased government support is needed.

**Contribution:**

More studies on costing will help in developing financial sustainability models for Africa.

## Background

Mobile health (mHealth) is the use of information technology (IT) such as short messaging service (SMS), telephone, or wireless data transmission in delivering health care.^1^ According to the World Health Organization’s (WHO) Global Observatory for eHealth survey conducted in 2009, mHealth implementation in high-income countries far surpassed that in low and middle-income countries. Africa had the lowest level of mHealth use at 47%.^2^ However, over the last decade, there has been an unprecedented rise in mHealth use across Africa, with some surveys showing it as high as 91% in South Africa and 80% in Nigeria. The use rate of mHealth was projected to rise even further to reflect the economic growth and the increased Internet penetration across Africa.^3^

Reviews have been published on different aspects of mHealth in developing countries covering topics such as the effectiveness of mHealth in non-communicable diseases, maternal and child health services, and infectious diseases.^4,5,6,7^ The main mHealth interventions used in the studies included in these reviews were SMS and phone calls. Furthermore, mHealth interventions have targeted behaviour change in developing countries. A systematic review has shown that social support techniques for reminder and medical appointment using SMS and phone calls are the most frequently used behaviour change interventions applied in mHealth.^4^ A major cross-cutting limitation is that these systematic reviews have been relevant in generating evidence on the role of mHealth in non-emergency settings only. In addition, they have not provided evidence on the use of telephone hotlines in both peace times and during outbreaks of infectious diseases in Africa.

Telephone hotlines have been used in both outbreak and non-outbreak settings to provide essential services in a simple way to the public.^8,9^ During outbreaks of infectious diseases, they have been beneficial as a means of communication with the public and to help in the surveillance of diseases like Ebola and COVID-19.^9,10^ In the wake of the COVID-19 pandemic, African countries set up telephone hotlines to assist in their outbreak response, and there have been calls post-COVID-19 for mHealth strategies to be scaled up and sustained in Africa.^11,12^ To scale up and sustain telephone hotlines, there is a need to understand the challenges encountered and to identify key areas of opportunities. Despite this need, the evidence on the barriers and facilitators to the sustainability of telephone hotlines for outbreaks of infectious diseases in Africa is lacking.

Because of this paucity of evidence on the use and sustainability of telephone hotlines in outbreak situations, we conducted this study to explore the barriers and facilitators for sustainability of telephone hotlines during infectious disease outbreaks. The study integrated a scoping review and qualitative component to ensure that a comprehensive analysis of the barriers and facilitators was provided. Importantly, the scoping review helped in identifying some of the authors in the countries who later agreed to be interviewed.

## Methods

A scoping review and qualitative methods using key informant online interviews were used in the study.

### The review

The review was conducted in accordance with the Preferred Reporting Items for Systematic review and Meta-analysis extensions for Scoping Reviews (PRISMA-ScR) guidelines for conducting scoping reviews.^13^

### Eligibility criteria

In this study, we included only those hotlines that were set up primarily for outbreaks or repurposed from an existing toll-free line or extended for use as part of an outbreak response. Articles were included if they mentioned the use of a telephone hotline in outbreak response and the barriers and/or facilitators in operating the hotline. Only hotlines in Africa were considered. There was no restriction on the language. Peer-reviewed publications and grey literature were included.

A telephone hotline for health was defined as a phone helpline that provides health information or responds to concerns raised by those calling. The hotline can either be a toll-free call line or charged at a fixed cost.^14^ Articles were excluded if they talked about outbreak response outside Africa or did not mention barriers and/or facilitators for the sustainability of hotlines used in outbreak response in Africa.

### Source of information and search strategy

Using medical subject headings (MeSH) and text key words, literature was searched relating to the use of telephone hotlines during outbreaks in Africa. The search was carried out on 30 June 2021 and updated on 30 April 2022. A final search was conducted on 30 September 2022. The search strategy was developed by research lead (N.F.) and refined with the help of London School of Hygiene and Tropical Medicine librarians. Ovid MEDLINE, Ovid Embase, Ovid Health and Psychosocial Instruments, PsycINFO, Global Health were searched by N.F. and a research assistant (A.N.). The search strategy used is shown in Figure 1-A1.

The outputs of the search using the databases above were exported to Mendeley desktop version 1.16.1, and duplicates were removed. After removal of duplicates, the titles and abstracts of the articles from the main database search were screened independently by N.F. and A.N. The full texts were obtained from the screened abstracts after inclusion and exclusion criteria were applied. Google and Google Scholar were searched for each of the 55 African countries to identify grey literature. The search terms used for Google and Google Scholar were: ‘telephone hotline or call centre’ and ‘hotline number’ and ‘name of country’. The hotline numbers were obtained using the African Union and United Nations International Children’s Emergency Fund (UNICEF) directories for hotline numbers. Grey literature articles were added to the peer-reviewed articles to give the total number of articles used in the review.

The reference lists of relevant full texts were searched to identify any relevant articles. After identifying the relevant articles, the authors were contacted for any information on other similar studies published on telephone hotlines in Africa.

### Data collection, data items

A data extraction spreadsheet was developed in Microsoft Excel version 2013. Data were extracted from the articles on items such as the main hotline operating requirements, sources of funding, barriers in setting up and operating the hotline, and facilitators for sustainability. Under the main hotline requirements, we were interested in knowing if the hotline was run virtually or in a physical centre, the expertise of the operators, and the availability of the services offered. Hotlines that were not toll-free were noted.

### Key informant interviews

#### Study setting, participants, and sample size justification

Telephone hotline implementers involved in infectious disease outbreak response in Africa were interviewed. After the scoping review search, the contact details of the peer-reviewed studies or grey literature authors were obtained. These authors were contacted for a semi-structured interview. After the interview, a snowball sampling was used in which the authors were asked to name colleagues in other countries that had experience using hotlines for outbreak response. The named colleagues were contacted directly through email to request an interview. In some countries, the participants also requested to be interviewed with other team members who have representation from the Ministries of Health and partner organisations that support telephone hotlines in-country. We planned to approach and interview at least one telephone hotline implementer from 55 African countries. However, saturation with repeating themes was reached at the point where 30 participants from 12 African countries were recruited and interviewed. These countries included Cameroon, the Democratic Republic of Congo (DRC), Uganda, South Sudan, Ghana, Nigeria, Ivory Coast, Sierra Leone, Malawi, Mozambique, Zambia, and Zimbabwe.

### Study design and data collection

One-on-one interviews and focus group discussions were conducted via Zoom between 15 July 2021 and 30 November 2021. The focus group discussions involved participants from Ministries of Health and implementing organisations in-country. The questions in the semi-structured questionnaire were targeted to explore the origin of the hotline, the purpose, the barriers and facilitators for sustainability.

After signing the consent form, the interviews were scheduled, and the questionnaire was sent at least 1 week before the day of the interview. The interviews were recorded and lasted between 45 min and 60 min. The audio recordings were stored in the principal investigators’ personal computer for analysis. The interviews were conducted by the principal investigator in French and English. One interview with the participant from Mozambique was conducted with the help of a consultant who was proficient in Portuguese. The principal investigator participated in the interview as an observer. The audio recordings of the interviews were transcribed into English and uploaded into NVivo version 12. Cases nodes and codes were generated, and the data for each country were classified and grouped into generalisable themes related to the challenges, mitigation strategies and opportunities of the hotlines by A.N. and cross-checked by N.F.

### Data analysis and synthesis

The conceptual framework for the data analyses is shown in Figure 2-A1.

In summary, emerging themes on barriers and facilitators for sustainability were derived from the interviews and the scoping review. The emerging themes on challenges were first grouped according to the regions in Africa and common major themes were further identified to present a coherent narrative.

Key phrases and expressions from the key informant interviews were used to support the findings.

### Ethical considerations

Ethics approval for the interviews was obtained from the Ethics Committee of the London School of Hygiene and Tropical Medicine (LSHTM) with reference number 25947. Before conducting any of the interviews, informed consent was obtained from all participants. A participant information sheet and consent form were shared with the participant, and after agreeing to participate in the study, the consent form was signed by both parties before starting the online interviews.

## Results

### General characteristics

Figure 1 is the PRISMA flow diagram showing the selection of studies in the scoping review. Out of 1153 non-duplicate articles from the database search, 11 peer-reviewed studies were included. These 11 studies were added to 14 grey literature articles from Google and Google Scholar search. A total of 25 articles were finally included. The list of references for articles is shown in Table 1-A1. The articles were from 20 African countries. These countries were in Northern Africa,^15,16^ West Africa,^17,18,19,20,21,22,23,24,25^ East Africa,^26,27,28^ Central Africa^10,29,30,31^ and Southern Africa^32,33,34,35,36,37,38^.

**FIGURE 1:**
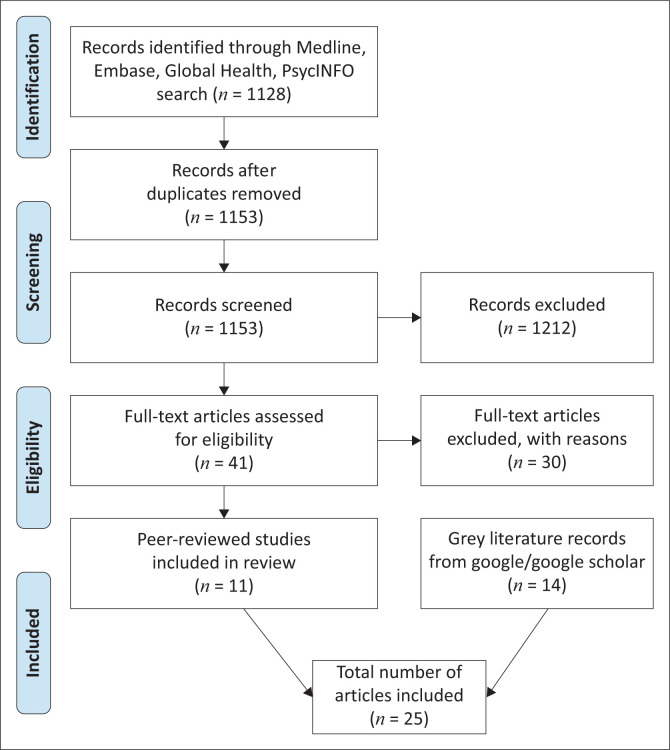
PRISMA flow diagram showing the selection of studies for the review.

Table 1 shows the general characteristics of the hotlines from the scoping review according to the regions in Africa. Additional information can be found in Table 1-A2. Most publications were from western Africa. The hotlines in the northern region were purely remote and hotline operators were given mobile phones to work from home. In South Africa, the hotline was both physical and remote. The hotlines in majority of central and western African countries relied on funding support from external sources.

**TABLE 1:** General characteristics of telephone hotlines from the scoping review.

Region in Africa and countries	Main hotline characteristics	Source of funding
Northern region (Algeria and Libya)	Virtual remote call centre. Operators were trained volunteers. Services available 24 h in a day for 7 days of the week.	From Ministry of Health for Algeria and external sources for Libya.
Eastern region (Kenya and South Sudan)	Physical call centre. Operators were medical doctors, medical students, and other health professionals. Services available 24 h in a day for 7 days of the week.	The government in the case of Kenya. External sources in the case of South Sudan.
Western region (Burkina Faso, Cote D’Ivoire, Ghana, Guinea, Liberia, Nigeria, Sierra Leone)	Physical call centre. Not toll-free in Burkina Faso and Cote D’Ivoire. Expertise of the operators were variable from untrained volunteers in Burkina Faso to skilled experts in Nigeria. Services available 24 h in a day for 7 days of the week.	Mixed support from government and external sources in Burkina Faso, Nigeria and Sierra Leone. Purely external support in Cote D’Ivoire, Guinea and Liberia.
Central region (Cameroon, Central African Republic, Chad, DR Congo)	Physical call centre. Operators were mostly healthcare workers. Services available 24 h in a day for 7 days of the week.	Mainly external support, except for Chad where the government provides the support.
Southern Africa (Madagascar, Malawi, Mozambique, Namibia, South Africa)	Physical call centres. Mix of physical and physical in South Africa Correction: A mix of physical and virtual in South Africa. Operators were healthcare workers. In South Africa, the services were available from 8:00 to 17:00 Monday to Friday.	Mixed support from government and external sources in Madagascar and Malawi. Purely external in Mozambique. Purely government in Namibia. Support from Doctors’ Association in South Africa.

Table 2 shows the summary of key informants interviewed from different countries. From 12 countries, 6 focus group discussions (with a total of 21 participants) and 6 individual interviews (with a total of 9 participants) were conducted.

**TABLE 2:** General characteristics of key informants interviewed.

Country	Key informant(s)
Cameroon	A total of four interviewed in the focus group discussion. All were staff from the Ministry of Health (MoH).
DR Congo	One person was interviewed. This was a senior hotline manager working with VillageReach and supporting the MoH in the DRC.
Ghana	Two people were interviewed separately. One senior and one junior manager of the hotline.
Ivory Coast	One person was interviewed. This person worked for an implementing partner supporting the hotline in developing a rumour tracking system.
Malawi	Three people interviewed in a focus group discussion. One person from MoH and the others from partner organisations like VillageReach supporting the MoH.
Mozambique	Two people interviewed separately. One person working with implementing partner and one senior manager from MoH who supervised the hotline.
Nigeria	Three people from the Nigerian CDC interviewed in a focus group discussion.
Sierra Leone	One person interviewed. This person worked during the Ebola outbreak and has detailed understanding about the hotline and changes over time.
South Sudan	Four people interviewed in a focus group discussion. One person from country CDC and the others from the MoH.
Uganda	Two people interviewed separately. They were from partner organisations supporting the MoH.
Zambia	Four people were interviewed in a focus group discussion. Two were from the MoH and two from the Zambia Public Health Institute (ZNPHI).
Zimbabwe	Three people were interviewed. Two in a focus group discussion and one person interviewed separately. All interviewees were from the MoH.

DRC, Democratic Republic of the Congo; CDC, Centers for Disease Control and Prevention; MoH, Ministries of Health.

### Hotline barriers

Table 3 and Table 4 show the telephone hotline barriers. The barriers were grouped into five themes: calls and data management; human resource; financial; technology and infrastructure; and social challenges. Additional details can be found in Table 1-A3. Table 3 presents the summary from scoping review, while Table 4 presents the summary from interviews. The tables complement each other with Table 4 being more elaborate by delving into more detailed comments from the participants interviewed.

**TABLE 3:** Hotline barriers and facilitators for sustainability (from scoping review).

Theme (barriers)	Examples of barriers under theme	Facilitators for sustainability
Calls and data management (most frequent)	High call volumes and long waiting times. Prank calls. Flawed government communication and case management team. Lack of standard operating procedures to verify, escalate and follow-up. Lack of reliable mechanism for reporting. Lack of training of volunteers on communication and handling calls. Security incidents hampered the follow-up of calls.	Government ownership. Integration of hotline into routine services such as surveillance, immunisation, and maternal health services. Private mobile telephone companies willing to support the hotline through corporate social responsibility mechanisms. Co-ownership by government and international organisations through public-private partnerships.
Human resource	Limited staff. Poor motivation of call centre operators. Competing responsibilities reducing engagement of clinicians. High staff turnover. High attrition rate from response team because of a lack of motivation and incentives.	
Financial	High initial investment cost. Time-bound engagement from national and international partners. Lack of sustainable funding. Huge dependence of external funding.	
Technology and infrastructure	Limited phone and internet coverage Lack of equipment at call centres Frequent software malfunctioning Lack of interoperability between software used by different hotlines in the country.	
Social	Language barriers. Lack of continuous social mobilisation. Lack of trust in the system. Misconception about the actual role of the hotline. Hotline number ‘6666’ fuelling religious misconceptions about hotline.	

**TABLE 4:** Hotline barriers and facilitators for sustainability (from interviews).

Theme (barrier)	Examples of barriers under theme	Opportunities for sustainability
Calls and data management (most frequent. Reported in 10 out of 12 countries during interviews)	Lengthy hotline number. Overwhelming number of calls. Use of third-party services to process calls. Absence of software in other hotlines. Disconnect between other hotlines and the national security hotline. Delays in integrating WhatsApp and IVR. Challenge in synchronising call and demographic data. Lack of referral and follow-up system. Callers are also referred to health facilities, but these facilities do not have any system in place to know and report back on these referrals. Number of digits of the hotline number too long. High call volumes leading to unattended calls. Insult to call centre agents. High burden on cost of calls. Prank calls. Long queue times. Inability to incorporate educational messages for callers while they waited in the queue. Lack of interactive voice recording. Inability to record calls. Inadequate or incompetent response by hotline operators to callers.	Government ownership in various ways: Hotline will be used for routine surveillance. Hotline will be used for hospital referrals. Hotline will be used for other purposes like maternal and child health. Hotline has been used for different purposes such as vaccine trial, psychosocial support. The hotline is used as main national emergency hotline by the government. Shared ownership with the government. The FDC to secure funding for the hotline for operations like technology, while the government provides the needed human resource. The hotline to be also used to target youth health issues using WhatsApp. Public-private partnerships with telecom companies to provide calls at no cost and other partnerships to support the government with the cost of running call centres. Partnership with private telecom companies and insurance companies to support remote services offered by the hotline with a small fee charge. Some telecom companies like Safaricom donated call centres to the government and covered for the cost. They also worked with the government to amplify signals and strengthen the network.
Human resource	Inadequate human resources and funding for salaries. In some cases, like in Malawi, 25 members of staff to handle 60 000 calls per week. Operators worked voluntarily with only transport remuneration. Substantial reduction in staff members because of financial constraints. Lack of incentives leading to suboptimal work from rapid response teams.	
Financial	Huge dependence on donor funding. Delays in recharge of call credit. Operators paying from their pocket for call credit. Limited funding for hotlines operators and night staff. Lack of financial incentives and salaries for volunteers.	
Technology and infrastructure	Lack of equipment and infrastructure in regional call centres. Poor network coverage and poor IT expertise. The web-based system sometimes crashed leading to manual data capture and difficulties in compiling electronic data. Malfunctioning of laptops because of overuse. Limited network coverage in some areas with no signals. Identifying and using the right software during the rainy season. Lack of office space and IT equipment. Low ownership of phones in rural communities. Absence of regulation of the telecommunication sector in Uganda. Absence of regulation of telemedicine. Difficulty in implementing necessary electronic updates during new outbreaks. The software used to triage is the manual version and easily breaks down. There is also a lack of dashboard for better data visualisation.	
Social	Lack of trust in the toll-free number. The idea of calling a toll-free number was new to the public, and the call centre had to educate people on its use, which was slow because of a lack of awareness. Need to have operators who spoke multiple languages. Conspiracy theories and stigma. Conspiracy theories about the biblical interpretation of the hotline number (6666). Callers asking to join sects for money. Hesitancy to use the hotline as a form of political opposition to the government. Lack of public awareness about the hotline’s existence. Need for a behaviour change to adapt to the new concept. Lack of funding for mass media awareness campaigns.	

IVR, interactive voice response; IT, information technology; FDC, Foundation for Community Development.

The most frequent barriers were associated with calls and data management. High call volumes brought the hotlines under substantial strain and quickly overwhelmed the system. The high number of prank calls and threats from callers compounded this strain. There was a lack of mechanisms such as automatic voice recording and interactive voice response (IVR) that could have facilitated the processing of calls, reduced response time and prevented the long queues of desperate callers. Inadequate responses were provided to callers because handling calls was challenging, and volunteers needed better communication skills.

Hotlines used in event-based surveillance lacked standard operating procedures for risk assessment, verification, and follow-up of escalated signals. In the DRC, the follow-up of escalated signals was constrained by ongoing insecurity, while in South Sudan, the resources to engage, motivate and monitor the response teams were limited. In Zimbabwe, the high casualties strained the response teams, which could not investigate all the community concerns. The communities, therefore, lost trust in the ability of the rapid response team to meet their needs.

One major human resource barrier was the limited number of staff that could have improved the ability of the hotline to respond to demand when the call volumes were high. In Malawi, despite the relatively easy transition to government ownership, there were just 25 staff members to respond to about 60 000 calls per week because of the high demand for COVID-19 information. Less than a fifth of these calls were answered and followed up. The hotlines used mainly volunteers that needed more motivation and incentives to keep them fully committed, resulting in high staff turnover. When qualified healthcare workers such as clinicians were involved in running the hotline, there was limited engagement because of competing responsibilities.^29^

Concerning the challenges of using volunteers and ensuring their sustained commitment, one respondent said:

‘… it was also difficult to find people to work for the hotline since it was during lockdown and there was no movement and also since it was a voluntary thing, we had to think how then do we identify these volunteers, how do we capacitate them, how do we schedule their work and how do we move them from their homes to the work place and back. How do we cover the midnight shift so it was those things.’ (> 50 years old, male, Hotline manager and nurse, Zimbabwe)

Financial barriers were the high initial investment cost, limited funds to support staff salaries, limited funding support from the government, and the considerable dependence on external donor funding, which was usually time-bound, unreliable and unsustainable. For example, in Ghana, financial support for operators was limited in the form of call credit, and the operators had to pay for the call credit. In addition, because of the limited financial support for staff salaries, the number of staff and the operational hours were reduced. Fewer number of staff working at night were made available to take calls. Concerning the dependence on donor funding, one respondent said:

‘… but the biggest challenge we faced is financing the hotline and how long will the country be able to support this because other hotlines have ended because of lack of funding and for example if a sponsor decide to stop funding the hotline, then the hotline will just end, so in the long run that is one of the biggest challenges the hotline might face.’ (< 40 years old, female, Hotline manger, VillageReach, DRC)

Technology and infrastructural barriers were linked to equipment, network infrastructure and IT software. A lack of essential equipment and poor maintenance of available equipment led to overuse and frequent malfunction of laptops and desktops. The software that was supposed to help in triaging calls was manually operated with frequent breakdowns. The phone network infrastructure was unreliable and poorly distributed, especially in remote areas. Even in urban areas where the network strength was expected to be strong, the reverse was the case because the hotline number was frequently offline and not accessible in time of need.

One respondent said:

‘Another challenge that I remember is in the districts a lot of people didn’t have call reception to call so we did create some call boxes that used solar panels or electricity you know like a pay phone but it is free and it is using a bigger antenna so we hired it in those areas that didn’t have cell connection and eventually we were able to start capturing calls that came from the districts that came into national number back to the district agents.’ (< 40 years old, female, Hotline health worker, Sierra Leone.)

Another respondent said:

‘So, we have upgraded the system and we have included a few more staff to enable the system to work on a 24hr basis as well as enabling the remote access that would allow people to attend work from home because of COVID restrictions. So, the lack of capability of moving forward and the limited resources in terms of equipment were some of the main challenges.’ (40 years old, male, Hotline supervisor, Mozambique)

Frequent malfunction of the software and web-based systems used in data collection and analysis was noted. There was also a lack of interoperability between different software used in a single country. In Ghana, where more than one hotline was used, the software used in the national security hotline was incompatible and linked with the others under the Ministry of Health. In South Sudan, the databases for the Ministry of Health and the WHO were running in parallel, leading to discrepancies in data registration, analysis and reporting.

Social barriers included the need for more awareness about the hotline, a lack of attention to social mobilisation, and distrust about the hotline’s purpose because of conspiracy theories, fake news, and sociocultural connotations. For example, in South Sudan, the number ‘6666’ paradoxically worsened the spread of conspiracies and misconceptions because it was perceived to have ‘demonic attributes’ and sparked fears that the hotline was an invitation to join sects.^27^ There was therefore a need to raise awareness geared at changing behaviour and ideologies about the role of the hotline. Raising awareness came with some difficulties.

One respondent said:

‘At the early stage the challenge was around awareness and mind shift. Awareness in the sense that people get to be aware of this platform that is available for them to utilize and there was no financial capacity for us to do that awareness in terms of radio and TV advert, or newspaper ads or any other form of media advert. Then also when there was awareness there was the challenge of mind shift. Ugandans were not used to using this kind of platform accessing health care workers on phone. So, the mind shift for people to trust remote health care access was difficult …’ (> 40 years old, male, IT experts and Hotline operator, Uganda)

### Facilitators for the sustainability of hotlines

Table 3 and Table 4 show the facilitators for sustaining telephone hotlines. The main facilitator for sustainability was the ownership by the government. Government ownership could be from the beginning of the hotline being set up by the Ministry of Health (MoH), or the hotline could be transitioned to the government by an organisation that helped set up and run the hotline. VillageReach set up and operated the hotline in Malawi, scaled up nationally and transitioned fully to the government. In Mozambique, there was shared ownership by the government and the Foundation for Community Development (FDC). This shared ownership is a form of public-private partnership that has reduced the pressure on the government because the FDC provided continuous support on the technology side, while the government provided the human resource to run the hotline. Concerning the public-private partnership, one respondent said:

‘… it has been successfully transitioned into the government as part of its activities. So, the MoH pays for the salaries of the workers but not for the infrastructure and equipment. Those are being paid through private partnership, network operators. The software maintenance is paid by FDC through global fund. The service is so well transitioned to the government that all maintenance is done in house so reducing the cost on external partners as the servers are already in the MoH and all those working on it are from the MoH.’ (> 40 years old, male, Hotline supervisor, Mozambique)

Government ownership also occurred in different ways. Some hotlines were fully integrated as part of the country’s public health emergency operating centre. This integration ensured the hotline could be repurposed and diversified for use in different outbreaks. Other hotlines were integrated as part of disease surveillance in the countries. Furthermore, when hotlines were not used for outbreaks, they were adapted to other government programmes such as immunisation, maternal and child health, nutrition, and public engagement with the youths in sexual and reproductive health and health promotion in schools.

The availability of partners committed to supporting the hotline was an essential enabler of sustainability. Partners such as the United States Centers for Disease Control and Prevention and the WHO and private sector organisations like PharmaAccess showed commitment to providing financial and technical support.

In Uganda, two business models were used. In one model, people were allowed to pay a small service charge for faster services. In another model, there was a collaboration with private insurance companies to ensure that people could benefit from better hotline services through their insurance coverage. Concerning the latter, one participant said:

‘… one major, private partnership that we have is the insurance companies and this has helped us to answer the question of sustainability especially during the pandemic and restrictions. There was a lot of dissatisfaction from the customers of private insurance because they were not able to utilize their insurance because they weren’t going to health facilities, yet they still were getting sick. So that is where we came in because this new system of hotline consultation brought a solution to this problem. These private companies jumped on the opportunity to provide access to health care to their customers without having to access medical facilities. Majority of these companies are the same ones in Kenya, Rwanda and Tanzania and therefore in East Africa we are the first partnership that private insurance has had to implement health care, or I would say the use of the hotline for medical service delivery ….’ (> 40 years old, male, IT expert and Hotline operator, Uganda)

## Discussion

There have been rapid advances in mobile phone technologies across the globe.^39^ As more people have access to mobile phones and Internet connection, mHealth technologies have increasingly been used to enhance outbreak response.^40^ The expansion in the use of mobile technologies such as telephone hotlines comes with challenges and opportunities. An understanding of the barriers and facilitators is critical in assisting hotline implementers and stakeholders develop solutions to overcome the barriers and develop strategies for sustainability. This study provides an in-depth assessment of the evidence on the challenges and opportunities for sustainability.

We found that the management of calls and data constituted a significant challenge for the hotlines. Other studies that have considered mHealth in general also found that technical challenges such as call management constituted a significant challenge.^41,42^ For hotlines to function effectively, the call operators must be able to receive calls, carry out registration of signals, assessment of signals, verification, reporting and follow-up of signals.^43^ The hotlines we found in our study lacked capacity to adequately implement these critical steps. Training of volunteers was therefore needed to optimise hotline operations. To overcome this barrier, volunteers were trained in call management and in communication. Some operators who spoke multiple languages were recruited. For some hotlines like in Mozambique, a system called ‘VIARO’ was put in place to help synchronise calls and data. In addition, WhatsApp was introduced to ensure effective feedback on referrals.

The hotlines encountered challenges with data collection because of software problems and the lack of interoperability. Interoperability uses standards, interfaces, and protocols to link systems using appropriate techniques and methodologies.^44^ Ndlovu et al. explored the interoperability challenges with mHealth in the Botswana healthcare system and found that there were different mHealth software systems from different vendors and donors in public and private health sectors, which were not interoperable resulting in fragmented care delivery, duplication of effort and unnecessary healthcare expenditure.^44^ Regarding telephone hotlines in outbreaks, this lack of interoperability in places like South Sudan led to inconsistencies in data collection, processing and analysis between partners and the MoH. When multiple data collection systems, flows and platforms are used within the healthcare system for surveillance, there is a lack of coherence and policy to guide data collection at different levels of the health system.^45^ In addition to donor requirements to collect data for monitoring and evaluating health initiatives, more complex challenges may result from the different data platforms used. To overcome these challenges in low- and middle-income countries (LMICs), the use of a system with defined data standards for mobile and computer-based platforms that are interoperable and open may be useful.^45^ These interoperable and open platforms may allow real-time data collection at the community level and reporting within health delivery facilities (including electronic medical recorders) to be linked directly to district health information systems for aggregation and access at the regional and national levels.

We found that using specific telephone hotline numbers that had perceived negative sociocultural interpretations can have a negative impact on the understanding of the role of the hotline, acceptability, uptake, and adoption. A study by O’Connor et al. that analysed the impact of contextual barriers in the implementation of mHealth technologies in Africa emphasised that unless there is a shift in sociocultural beliefs held by some communities, the adoption of such technologies will be in jeopardy.^46^ Sociocultural factors constitute the corner stone of the acceptability of any mHealth intervention. These factors are a set of beliefs and norms that individuals in a society hold consciously or unconsciously.^47^ The sociocultural factors are intricately linked to cognitive and political factors. Social norms are extremely important in LMICs because they are driven by collectivist strategies, which contrasts with more individualistic strategies in developed countries.^48^ In our study, a lack of consideration for social dynamics negatively affected community’s trust in the government and implementing partners. To overcome social barriers, there was widespread advertisement and social mobilisation to increase awareness and trust about hotlines.

One of the key infrastructural and technological challenges we found in our study is the poor network coverage across many countries. Approximately 47% of the global population in developing countries resides in rural areas.^49^ These rural populations face significant power outages and struggle to cope with telephone services. The lack of network services may hamper the delivery of mHealth services.^50^ Taking Malawi as an example, the situation was made worse because of limited mobile network providers which increased the cost of using telephones.^50^ Other studies have confirmed that basic infrastructure is indispensable and remains a major challenge in the implementation of mHealth interventions in developing countries.^51,52,53^ Key facilitators to overcome the infrastructural barriers were noted. In Sierra Leone, the call centre had to put in place call booths powered by solar panels for toll-free calls to improve their coverage. Some telecom companies like Safaricom donated call centres and partnered with the government to amplify signals and strengthen the network coverage. In Malawi, VillageReach and the MoH had a public-private partnership with the mobile network operator Airtel, to amplify the signal strengths in remote areas during the scale up phase of the telephone hotline.

Finally, even though telephone hotlines, like any other mHealth interventions, initially benefit from start-up funding from government and donors, the challenge of financial sustainability and who ultimately pays pose significant barriers.^54^ Our study found that there is still a high dependence on external funding, which may only sometimes be guaranteed. Paying staff salaries, capacity building for staff, and purchasing and maintaining equipment and software constitute some of the essential components of the running cost of telephone hotlines. Transitioning to government ownership is ideally expected to provide crucial financial sustainability; but given the limited resources, governments may need to prioritise other interventions over hotlines. For example, there might need to be more government staff to support the hotline when they have other competing priorities. These financial challenges were also captured in the mHealth Alliance report on sustainable financing for mobile health in LMICs.^54^ Exploring the components of the hotline that can be taken up by commercial forces and how donors can complement government investment are critical questions that will need to be answered when developing and expanding on the models of financial sustainability.^54^

From this study, the main facilitator for the financial and human resource sustainability of the hotlines were the different forms of government ownership. Complete government ownership would be ideal; but given the limited resources and other competing priorities, a shared ownership with partners may be the way forward. Dharmayat et al. assessed the sustainability of mHealth interventions in sub-Saharan Africa using Malawi as a case study. Participants emphasised on the key role of the MoH in ensuring sustainability but also noted that a myriad of factors such as engagement and cooperation within government departments, between the government and external funding agencies, local non-government organisations and other partners, will influence the long-term sustainability of programmes.^55^ In our study, such partnerships between the Ministries of Health, funders and key stakeholders were noted.

### Strengths and limitations of the study

One limitation of this study was that there was no response from North African countries to be interviewed. However, despite interviewing 30 participants from 12 out of 55 African countries (mainly from sub-Saharan Africa), the sample size was justified because saturation was reached. Despite this limitation, our study provides relevant qualitative finds with views from hotline operators on the barriers and facilitators for telephone hotlines in the setting of outbreaks in Africa. Even though there was no participant interviewed from North Africa, the scoping review enabled us to obtain some data from Northern African countries. The evidence from this study will be essential to policy makers, funders and implementers of hotlines by enabling them to understand how to optimise the functions of telephone hotlines and ensure their sustainability during and post outbreaks.

## Conclusion and recommendations

Telephone hotlines face significant barriers in their implementation. However, opportunities exist that help overcome the barriers and facilitate the sustainability of the hotlines. Building the capacity of operators in hotlines is critical. Financial barriers were frequently mentioned in the interviews, but limited published data on costing are available in the literature. While there is need for more government support and ownership, future costing and cost-effectiveness studies are needed to provide a clearer picture on the financial implication of hotlines including the start-up and running costs. These costing studies will provide additional data on the value of hotline and will help develop sustainability models applicable in Africa.

## Figures and Tables

**FIGURE 1-A1:**
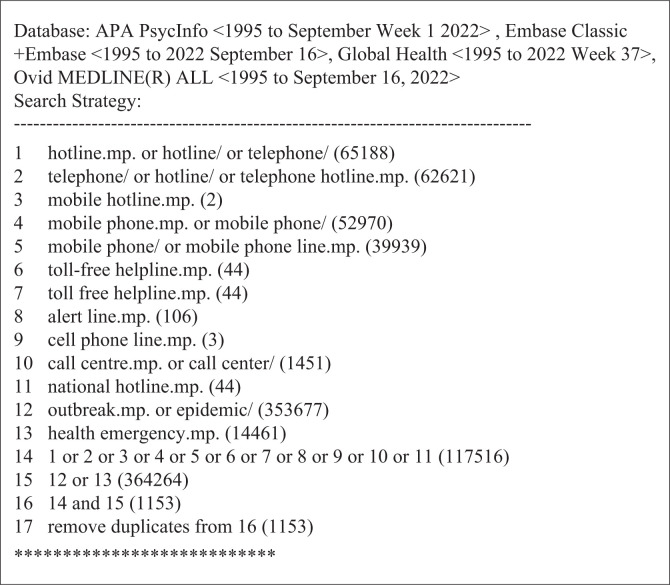
Database search strategy.

**FIGURE 2-A1:**
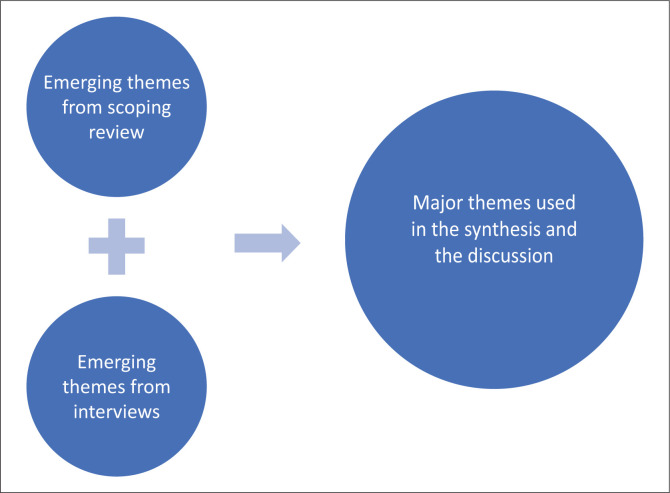
Conceptual framework used in the data synthesis.

## Appendix 1

**TABLE 1-A1:** List of references of articles from the scoping review.

Country	References
Algeria	Algeria’s COVID-19 hotline props up rapid response |WHO| Regional Office for Africa
Burkina Faso	https://sparc.africa/2021/06/strategic-investments-in-communication-improve-the-fight-against-covid-19-the-case-of-the-3535-helpline-in-burkina-faso/
Cameroon	Strengthening timely detection and reporting of unusual respiratory events from health facilities in Yaoundé, Cameroon. Influenza and Other Respiratory Viruses.^29^
Central African Republic	https://reliefweb.int/report/central-african-republic/around-clock-communication-fighting-covid-19-1212-call-centre
Chad	Use of Call Centers in Polio Eradication Efforts in Island Settlement in Chad. *Journal of Immunological Sciences*.^30^
Cote D’Ivoire	https://www.aciafrica.org/news/1046/call-centre-offering-psychological-support-for-covid-19-cases-functional-in-ivory-coast Real-time tracking of COVID-19 rumours using community-based methods in Côte d’Ivoire. Global Health: Science and Practice.^19^
Democratic Republic of Congo (DRC)	Evaluation of Early Warning, Alert and Response System for Ebola Virus Disease, Democratic Republic of the Congo, 2018–2020. Emerging infectious diseases.^10^
Ghana	https://yen.com.gh/151549-coronavirus-over-1-million-prank-calls-rocks-hotline-112.html
Guinea	Evaluation of a national call centre and a local alerts system for detection of new cases of Ebola virus disease – Guinea, 2014–2015. Morbidity and Mortality Weekly Report.^25^
Kenya	https://nation.africa/kenya/news/safaricom-20-000-call-covid-19-helpline-daily-286534
Liberia	https://time.com/3462090/liberia-ebola-hotline/
Libya	https://www.wfp.org/stories/coronavirus-hotline-libyans-inundated-calls
Madagascar	Madagascar COVID 19 sitrep 6–29 July 2020final.docx (unicef.org)
Malawi	https://skoll.org/2020/07/30/answering-the-call-how-villagereachs-covid-411-campaign-is-fighting-the-pandemic-with-innovative-mobile-solutions/
Mozambique	https://ccp.jhu.edu/2020/10/07/covid-call-center-mozambique/ https://clubofmozambique.com/news/covid-19-mozambiques-alo-vida-telephone-line-answers-questions-about-the-disease-listen-15949
Namibia	https://www.namibian.com.na/200346/archive-read/Toll-free-number-gets-360-000-calls
Nigeria	Evaluation of national event-based surveillance, Nigeria, 2016–2018. Emerging Infectious Diseases.^22^
Sierra Leone	The 117 call alert system in Sierra Leone: from rapid Ebola notification to routine death reporting. BMJ Global Health.^24^ National reporting of deaths after enhanced Ebola surveillance in Sierra Leone. PLoS Neglected Tropical Diseases.^23^
South Africa	https://isqua.org/latest-blog/covid-19-doctors-on-call-national-program-in-south-africa.html Effectiveness of 24-h mobile reporting tool during a malaria outbreak in Mpumalanga Province, South Africa. *Malaria Journal*.^33^
South Sudan	Analyses of the performance of the Ebola virus disease alert management system in South Sudan: August 2018 to November 2019. PLoS Neglected Tropical Diseases.^28^ Lessons learned from implementation of a national hotline for Ebola virus disease emergency preparedness in South Sudan. Conflict and Health.^27^

COVID-19, coronavirus disease 2019; WHO, World Health Organization.

**TABLE 2-A1:** General information about the hotlines.

Country	Origin and history	Hotline requirements	Hotline operators	Role of mobile network operators (MNOs)	Source of funding (and changes over time)
Algeria	Previously no dedicated hotline for outbreaks. New hotline (3030) was set up only for COVID-19.	Virtual/remote call centre. Operators worked from home and were in contact via Microsoft Teams to exchange information.	A total of 20 volunteers who were doctors. Training was offered but duration was unknown.	Not stated	Supported only by the Ministry of Health (MoH).
Burkina Faso	The first hotline set up by the government was overwhelmed within 2 weeks. The government then set up the 3535 hotlines from scratch for COVID-19. The first hotline was only available to callers in the capital city and the calls were not toll-free.	Physical call centre. Operators equipped with computers and telephones in a dedicated space.	Volunteers. Initially there was no training offered. The USAID stepped in to support the training of 141 call operators in management of calls and alerts, and communication.	Yes. Helped the government in setting up the hotline.	The government provides the salaries of the hotline’s operators. Operators were paid $10.00 per day. Other partners like the USAID provided training and equipment.
Cameroon	The government set up the 1510 from scratch as part of the incident management system activation at the Public Health Emergency operations centre. The 1510 was temporarily unavailable in April 2020 but services were restored. The 1511 was later set up for psychosocial support for the pandemic.	Physical call centre. Operators equipped with computers and telephones in a dedicated space. The hotline is decentralised to 4 regions that are hotspots for the COVID-19 pandemic handling 20 calls simultaneously.	MoH staff and volunteers temporarily redeployed. No clear information on the kind of training received.	Yes. Provided the toll-free lines.	The MoH and the US CDC. The US CDC was permanently supporting a hotline for respiratory illness that was repurposed for COVID-19.
Central African Republic	The hotline (1212) was set up for Ebola and repurposed for COVID-19.	Physical call centre. Operators equipped with communications equipment, computers and telephones in a dedicated space.	Five volunteers. Some had background in pharmaceutical engineering. Training was offered on how to handle large call volumes.	Yes. Provided connectivity services.	World Food Programme (WFP). Transitioned and managed by the MoH.
Chad	The hotline that was used for polio was never scaled up or repurposed. A new hotline for COVID-19 (the 1313) was set up.	Physical call centre. Operators equipped with communications equipment, computers and telephones in a dedicated space.	Not stated	Yes. Supported the government in setting up the hotline.	By the Ministries of Telecommunications.
Cote D’Ivoire	Hotlines (143, 144 and 101) were used for the Ebola response. These hotlines were repurposed for the COVID-19 response. Only the main hotline was toll-free.	Physical call centre. Operators equipped with communications equipment, computers and telephones in a dedicated space. The DHIS2 platform was used in coding rumours and handling the data.	A total of 64 operators. The level of expertise was unknown. Training on rumours was offered.	Yes. Helped making the main national hotline toll-free.	USAID through the Johns Hopkins Center for Communication Programs.
Democratic Republic of Congo (DRC)	Two hotlines existed for Ebola. There is no evidence that the Ebola hotline was converted to the COVID-19 hotline. Instead, a new hotline was set up with the support of VillageReach (the NGO that set up the Health Centre by Phone in Malawi).	Physical call centre. Operators equipped with communications equipment, computers and telephones in a dedicated space.	Healthcare workers. Number not stated. Training offered.	Yes. To amplify signals and support other functions like WhatsApp.	The government of DRC and other external funders such as UNICEF, Gavi, Bill and Melinda Gates.
Ghana	Hotlines set up for Ebola and cholera and general emergencies. Another hotline for national security. These were repurposed for COVID-19.	Physical call centre. Operators equipped with communications equipment, computers and telephones in a dedicated space.	Volunteers. Medical doctors and nurses trained.	Yes. To help in setting up the hotline and making it toll-free.	The Government.
Guinea	There was a hotline that was set up and used for the Ebola response in 2014. A new hotline was set up for COVID-19.	Physical call centre. Operators equipped with communications equipment, computers and telephones in a dedicated space.	Volunteers. Level of expertise unknown.	Yes. Helped in setting up the hotline and amplifying signals.	The Ebola hotline was supported by the US CDC. The source of funding for the COVID-19 hotline was not stated.
Kenya	A new hotline was set up from scratch for COVID-19.	Physical call centre. Operators equipped with communications equipment, computers and telephones in a dedicated space.	General hotline: volunteers and 300 medical doctors. Kisumu hotline: medical students.	Yes	The government and the local county department of health.
Liberia	The main hotline (4455) is an Ebola hotline that was repurposed for the COVID-19 response.	Physical call centre. Level of equipment not stated.	Ebola: University students and other volunteers. COVID-19: volunteers.	Not stated.	USAID
Libya	One main hotline which was used for humanitarian purposes by the WFP. This hotline was also used for COVID-19.	Remote call centre. Lack of internet and phone coverage at the call centre. The four hotline operators were given mobile phones to work from home.	Volunteers. More technical cases were redirected to clinicians available online.	Yes	Government of Luxembourg and the World Food Programme.
Madagascar	The two hotlines for COVID-19 (the 910 and 913) were set up from scratch. There is a hotline for routine sentinel surveillance for influenza-like illness.	Not stated.	Volunteers. For influenza-like illness hotline, sentinel general practitioners were the volunteers.	Not clear.	UNICEF for the COVID-19 hotlines. The government for the ILI hotline.
Malawi	Two hotlines existed for Ebola. Before COVID-19, a new hotline called the health centre by phone (CCPF) was set up and scaled up nationally. This hotline was adapted for use in COVID-19.	Physical call centre. Operators equipped with communications equipment, computers and telephones in a dedicated space.	Nurses, health care workers. More technical cases were referred to doctors online. Training offered in various aspects.	Yes. Helped in making the hotline toll-free and amplified signals.	Initially by GIZ, USAID and Gates Foundation. Now by the government.
Mozambique	The Alovida is the main hotline and was set up 21 years ago and was mainly used to respond to HIV or AIDs and Malaria. This hotline was extended for use in COVID-19.	Physical call centre. Operators equipped with communications equipment, computers and telephones in a dedicated space.	Nurses, health care workers. More technical cases were referred to doctors online. Training offered in various aspects.	Yes. Helped in making the hotline toll-free and amplified signals.	USAID through the Johns Hopkins Center for Communication Programs.
Namibia	The hotlines were set up from scratch to respond to COVID-19.	Not stated.	Medical students and health care professionals. A total of 45 staff.	Not stated.	The government.
Nigeria	Main hotline for the Nigerian CDC was set up before the COVID-19 pandemic. This was also used for COVID-19. There are other hotlines in different regions of the country.	Physical call centre. Operators equipped with communications equipment, computers and telephones in a dedicated space.	Nigerian CDC experts and volunteers.	Not stated.	The first hotline for integrated disease surveillance was funded by the University of Maryland.
Sierra Leone	The 117 national hotline was repurposed for use in the Ebola response as part of the emergency operations centre in 2014. After the outbreak, the hotline was used in reporting and surveillance for mortality. During COVID-19, the hotline was repurposed for the COVID-19 response.	Physical call centre. For Ebola, an internet database accessible in the districts where cases originated was set up. Direct software links with the district teams was possible. There is also a messaging system and voice calls are used.	Ebola: staff working with the e-Health Africa (an NGO). For COVID-19: volunteers	Not stated	The government with support from WHO and the US CDC.
South Africa	A malaria hotline was set up and is also functional till date. During the COVID-19 pandemic, there was a general national COVID-19 hotline.	Mix of physical and virtual. For the COVID-19 hotline, the calls were received by a local call centre and rooted to a doctor-on-call. They were helplines operated virtually with no need of a physical call centre. The COVID-19 hotline has WhatsApp integrated and an app for referrals.	440 volunteer doctors for the COVID-19 hotline.	Not stated	Doctors’ Association.
South Sudan	The main hotline is the 6666 that was used for Ebola and then repurposed for COVID-19.	Physical call centre. Even though it was part of the public health emergency operations, it was just stated that the operators were given cellular phones in their operation 24/7.	MoH employees. Several rounds of training on surveillance operational procedures, documentation, reporting, and risk communication.	Yes. Ensured the hotline was toll-free for callers.	WHO and the US CDC.

MoH, Ministry of Health; USAID, United States Agency for International Development; CDC, Centers for Disease Control and Prevention; NGO, non-governmental organisation; ILI, influenza like illnesses; GIZ, Germany Agency for International Cooperation; CCPF, Chipatala Cha Pa Foni; HIV, human immunodeficiency virus; AIDS, acquired immunodeficiency syndrome.

**TABLE 3-A1:** Challenges and mitigation strategies (from interviews).

Country	Key informant(s)	Challenges	Mitigating strategies
Cameroon	A total of four interviewed in the focus group discussion. All were staff from the MoH.	Social: Lack of trust in the toll-free number. Human resource: inadequate human resources and funding for salaries. Infrastructural: Lack of equipment and infrastructure in regional call centres.	Not stated
DR Congo	One person was interviewed. This was a senior hotline manager working with VillageReach and supporting the MoH in the DRC.	Call management: Lengthy hotline number. Overwhelming number of calls. Use of third-party services to process calls. Human resource: shortage of call centre operators. Financial: Huge dependence on donor funding. Response and follow-up: Challenges with timely intervention and follow-up of referrals from hotline. Field teams could not be easy contacted.	A new three-digit number was introduced for the hotline.
Ghana	Two people were interviewed separately. One senior and one junior manager of the hotline.	Infrastructure and IT: poor network coverage and poor IT expertise. Data collection and analytics: absence of software in other hotlines. Disconnect between other hotlines and the national security hotline. Call management: prank calls. Human resource: inadequate human resources. Financial: delays in recharge of call credit. Operators paying from their pocket for call credit.	Not stated
Ivory Coast	One person was interviewed. This person worked for an implementing partner supporting the hotline in developing a rumour tracking system.	Data collection and analytics: difficult systematic documentation when tracking misinformation.	Not stated
Malawi	Three people interviewed in a focus group discussion. One person from MoH and the others from partner organisations like VillageReach supporting the MoH.	Data collection: The web-based system sometimes crashed leading to manual data capture and difficulties in compiling electronic data. Call management: Only 15% – 17% of calls were handled because of high demand for COVID-19 information. Prank calls and people who called to insult operators. Staff had limited information at times to provide to callers. Human resource: 25 members of staff to handle 60 000 calls per week.	Not stated
Mozambique	Two people interviewed separately. One person working with implementing partner and one senior manager from MoH who supervised the hotline.	Call management: Delays in integrating WhatsApp and IVR. Challenge in synchronising call and demographic data. Human resource: Limited number of staff. Financial: Limited funding for hotlines operators and night staff. Follow-up: Lack of referral and follow-up system. Callers are also referred to health facilities but these facilities do not have any system in place to know and report back on these referrals. Infrastructure: Lack of equipment and infrastructure.	A system called VIARO is being put in place to help synchronise calls and data. WhatsApp introduced to ensure effective feedback on referrals.
Nigeria	Three people from the Nigerian CDC interviewed in a focus group discussion.	Infrastructure and IT: Malfunctioning of laptops because of overuse. Call management: Number of digits of the hotline number too long. High call volumes leading to unattended calls. Insult to call centre agents. High burden on cost of calls.	There was training of more volunteers and partnering with other call centres to handle more calls. The malfunctioning of laptops because of overwork was solved by the provision of sophisticated desktops during COVID-19.
Sierra Leone	One person interviewed. This person worked during the Ebola outbreak and has detailed understanding about the hotline and changes over time.	Infrastructure and IT: Limited network coverage in some areas with no signals. Identifying and using the right software during the rainy season. Lack of office space and IT equipment. Social challenges: The idea of calling a toll-free number was new to the public, and the call centre had to educate people on its use, which was slow because of lack of awareness. Need to have operators who spoke multiple languages.	The call centre had to put in place call booths powered by solar panels for toll-free calls. This improved call centre coverage. There was widespread advertisement and social mobilisation to increase awareness about the hotline. Some operators who spoke multiple languages were recruited. Some calls were transferred to these operators.
South Sudan	Four people interviewed in a focus group discussion. One person from country CDC and the others from the MoH.	Lack of government ownership and support: Absence of government ownership. Lack of financial, material and logistical support from the government. Infrastructural and IT: Network coverage and accessibility issues. Poor network coverage in the country. Low ownership of phones in rural communities. Social challenges: conspiracy theories and stigma. Conspiracy theories about the biblical interpretation of the hotline number (6666). Callers asking to join sects for money. Hesitancy to use the hotline as a form of political opposition to the government. Hotline callers who call to ask for money and those who insult call handlers. Data collection and analytics: Absence of an electronic data collection system Data collected using Excel sheets and paper. Limited use of data for decision making	Not stated
Uganda	Two people interviewed separately. They were from partner organisations supporting the MoH.	Infrastructure and IT: Absence of regulation of the telecommunication sector in Uganda. Absence of regulation of telemedicine. Difficulty in implementing necessary electronic updates during new outbreaks. Social challenges: Lack of public awareness about the hotline’s existence. Need for a behaviour change to adapt to the new concept. Lack of funding for mass media awareness campaigns. Data collection and analytics: Lack of interoperability with other public health services because of the use of different, incompatible software (such as Clinic Masters and MRS). Call management: Difficulty in handling high volume of calls.	Not stated
Zambia	Four people were interviewed in a focus group discussion. Two were from the MoH and two from the Zambia Public Health Institute (ZNPHI).	Infrastructure and IT: The software used to triage is the manual version and easily breaks down. There is also lack of dashboard for better data visualisation. Call management: Prank calls. Long queue times. Inadequate number of call agents. Inability to incorporate educational messages for callers while they waited in the queue.	The hotline worked with Telecom service providers to block the prank call numbers while more call centre agents were recruited and trained to meet demand.
Zimbabwe	Three people were interviewed. Two in a focus group discussion and one person interviewed separately. All interviewees were from the MoH.	Data collection and analytics: Limited data collection tools. Loss of data during manual collection by agents. Call management: Prank calls. Lack of interactive voice recording. Inability to record calls. Inadequate or incompetent response by hotline operators to callers. Human resource: Operators worked voluntarily with only transport remuneration. Substantial reduction in staff members because of financial constraints. Lack of incentives leading to suboptimal work from rapid response teams. Financial constraints: Lack of financial incentives and salaries for volunteers. Response and follow-up: Overwhelming COVID-19 casualties putting a strain on rapid response teams. Unable to investigate all community concerns from the call centre. Communities losing trust in the rapid response team’s ability to meet their needs.	

MoH, Ministries of Health; DRC, Democratic Republic of the Congo; IT, information technology; CDC, Centers for Disease Control and Prevention; IVR, interactive voice response; MRS, medical records systems.
